# Clinical Study on the Efficacy of Bevacizumab in Combination with Pembrolizumab on Cellular Immune Function in the Treatment of Driver Gene-Negative Stage IV Lung Adenocarcinoma

**DOI:** 10.1155/2022/7298192

**Published:** 2022-11-04

**Authors:** Jun Gong, Juanjuan Gu, Lili Jiang, Dongxia Zhao, Lili Shao, Xi Chen, Jianhua Liu

**Affiliations:** ^1^Department of Oncology, Tumor Hospital Affiliated to Nantong University, Jiangsu, China; ^2^Department of Oncology, Nantong Haimen District People's Hospital, Jiangsu, China

## Abstract

**Purpose:**

To explore the efficacy of bevacizumab in combination with PD-1 immune drug pembrolizumab on cellular immune function in the treatment of driver gene-negative stage IV lung adenocarcinoma and its short-term survival effect.

**Methods:**

From February 2020 to December 2021, 85 patients with driver gene-negative stage IV lung adenocarcinoma were admitted to our hospital and treated with first-line therapy, and their clinical records were reviewed retrospectively. According to the treatments, the patients were separated into two groups the combination group (*n* = 45) and the control group (*n* = 40). The treatment regimen of the control group was an AP chemotherapy regimen (pemetrexed combined with cisplatin) + PD-1 immune drug pembrolizumab. The treatment regimen of the combination group was AP chemotherapy regimen + PD-1 immune drug pembrolizumab combined with bevacizumab. We evaluated the pre- and post-treatment cellular immunological function of the two patient groups and discussed the difference between them.

**Results:**

There was a substantial difference in the overall effective rate and the disease control rate between the two groups, with the former being 27.50% compared to 48.89% and the latter being 72.50% compared to 93.33% among these 85 patients studied. The KPS for the combination group improved and stayed at 91.11% after treatment, which is considerably better than the KPS for the control group, which was 42.50% (*χ*^2^ = 23.09, *P* < 0.05). There was no significant difference (*P* > 0.05) in the numbers of CD3+, CD4+, CD19+, CD8+, or CD4+/CD8+ cells pretreatment between the two groups, but after treatment, the combination group had significantly higher numbers of all these cells. Neither the CD8+ nor the CD19+ level was significantly different between the control and combination groups (*P* > 0.05). Furthermore, the incidence of common clinical side effects was similar between the two groups (*P* > 0.05). Proteinuria, tiredness, increased alanine aminotransferase, hypertension, immunological pneumonia, muscle pain, arthralgia, hypothyroidism, etc. were the most common side effects reported among both groups throughout therapy. A grade IV side effect is rare. After follow-up until March 2022, the median PFS for the control group was 9.00 ± 1.65 months (95% CI, 5.76–12.24) and the mean PFS was 11.48 ± 0.91 months (95% CI, 9.69–13.26). Comparison of the median PFS of the combination group (13.00 ± 1.10) months (95% CI: 10.84–15.16) with the average PFS of the group (15.52 ± 0.88) months (95% CI = 13.79–17.25) reveals a statistically significant difference (*P* < 0.05).

**Conclusion:**

Combining bevacizumab with the PD-1 immune medication pembrolizumab to treat patients with stage IV lung adenocarcinoma improves the quality of life, short-term therapeutic effectiveness, immune function, and PFS.

## 1. Introduction

Lung cancer has the highest fatality rate of all malignant tumors in China and is among the most lethal cancers worldwide. Nearly 40% of all lung cancer patients are caused by lung adenocarcinoma, making it the most common pathological subtype of lung cancer [1, 2]. Most patients are diagnosed in the middle to late stages of lung adenocarcinoma, long after the optimal time for surgical treatment has passed, due to factors such as nonspecific clinical symptoms and the lack of knowledge in the general population. Statistics show that just 15% to 19% of stage IV patients will survive for a full year [3]. According to relevant research results, after lung adenocarcinoma develops into stage IV, its progression of invasion and metastasis cannot be curbed, and its resistance and sensitivity to conventional chemotherapy drugs are low [4], making it difficult to treat.

There are two main categories in which individuals with advanced lung adenocarcinoma fall when it comes to therapeutic therapy. One is for patients with positive driver genes, whose first-line treatment is targeted therapy. At present, for stage IV driver gene-positive NSCLC, there are three most important targets such as EGFR mutations, ALK fusions, and ROS1 fusions. The other category is driver gene-negative patients, whose primary treatment is platinum-based chemotherapy, combined with antivascular or immunotherapy.

PD-1 immunotherapy has attracted interest as the latest treatment method for tumors in recent years. With more and more research on tumor-related immunotherapy, the treatment of NSCLC has been promoted into the era of immunotherapy. Some individuals have shown prolonged survival after receiving 1/PD-L1 inhibitors. In 2019, the results of the KEYNOTE-00's 5-year follow-up were presented at the annual conference of the American Society of Clinical Oncology (ASCO). Survival rates at 5 years were 29.6% for those whose PD-L1 TPS was ≥ 50% or below. The KEYNOTE-024 trial was the first to show that first-line PD-1 monoclonal antibody monotherapy in EGFR/ALK wild-type, PD-L1 TPS 50% of newly treated advanced NSCLC patients showed significantly enhance overall survival relative to chemotherapy. Patients with advanced NSCLC with PD-L1 expression of at least 50% are now eligible for first-line pembrolizumab therapy, as recommended by the Food and Drug Administration (FDA) of the United States.

Additionally, it has been found that immune checkpoint blockade therapy may be effective in driver gene-negative cases [5], and PD-1 immunotherapy has been found to achieve significant efficacy in driver gene-negative lung adenocarcinoma patients, especially in stage IV patients. The complexity and dynamics of the tumor microenvironment [6] may explain why only a subset of patients responds to PD-1 antibody treatment. Since angiogenesis is linked to immunosuppression in the tumor microenvironment, facilitating the immune escape of tumor cells, antiangiogenic treatment may boost anti-PD-L1 therapy by bolstering vascular alterations [7]. Finally, the sensitivity of antiangiogenic treatment can be enhanced and its effectiveness can be maintained by adding anti-PD-1 therapy. Monoclonal antibody bevacizumab suppresses tumor development by preventing human vascular endothelial growth factor (VEGF) from attaching to its receptors on the surface of vascular endothelial cells. This in turn reduces the formation of new blood vessels, which in turn slows tumor growth [8]. It also has the effect of normalizing tumor blood vessels, which is conducive to the entry of chemotherapeutic drugs into tumor tissue to better play the antitumor effect [9]. Therefore, in this study, patients with stage IV lung adenocarcinoma who lacked a driving gene were prioritized for therapy. Treatment groups were compared between those receiving chemotherapy as part of the AP regimen and those receiving immunotherapy with pembrolizumab. Clinical use of bevacizumab in combination with pembrolizumab for the treatment of patients with advanced driver gene-negative lung adenocarcinoma should take into account variations in short-term therapeutic efficacy, quality of life, immune function, and survival time.

## 2. Material and Method

### 2.1. Data Selection

The clinical data of 85 patients with driver gene-negative stage IV lung cancer treated with first-line therapy in our hospital from February 2020 to December 2021 were analyzed. The treatment of the control group was an AP chemotherapy regimen (pemetrexed combined with cisplatin) + pembrolizumab treatment. The treatment of the combination group was AP chemotherapy regimen + bevacizumab + pembrolizumab.

### 2.2. Inclusion and Exclusion Criteria

#### 2.2.1. Inclusion Criteria

The inclusion criteria were as follows:

1. Stage IV NSCLC (driver gene-negative stage IV lung adenocarcinoma) confirmed by pathology
2. PD-L1 tumor proportion score ≥ 1%
3. The patient has not used immunosuppressive agents and bevacizumab in the past treatment
4. The estimated survival period is greater than 3 months

#### 2.2.2. Exclusion Criteria

The exclusion criteria were as follows:

1. Patients having a previous diagnosis of pulmonary fibrosis or pulmonary interstitial illness
2. Those with a history of infusion reactions to other antibody therapy
3. Those suffering from autoimmune diseases
4. Patients with severe cardiovascular disease.

### 2.3. Treatment Methods

#### 2.3.1. Control Group

Immunosuppressant: pembrolizumab (trade name: Kerida, Merck, import drug registration number: S20180019) 200 mg/time, on the 1st day, intravenous infusion for 1 h, once every 3 weeks. A chemotherapy regimen (AP regimen): pemetrexed 500 mg/m2. Day 1, intravenous infusion, cisplatin 75 mg/m2; day 1–3, intravenous infusion for 1 hour and 1 chemotherapy every 3 weeks cycle. Chemotherapy was administered for 4 to 6 cycles according to patient benefit and tolerance. Then every 3 weeks on the first day, the PD-1 inhibitor was instilled until disease progression or intolerable adverse reactions.

#### 2.3.2. Combination Group

On the basis of the treatment of the control group, bevacizumab (Roche Diagnostics GmbH, registration number S20170036) was added for treatment. On the first day, bevacizumab 7.5 mg/kg, intravenous drip for 1 h. Combination therapy was repeated every 3 weeks. Then every 3 weeks on the 1st day, the PD-1 inhibitor was instilled until disease progression or intolerable adverse reactions.

### 2.4. Indicator of Treatment Response

#### 2.4.1. Evaluation of Short-Term Treatment Efficacy

In this study, treatment effectiveness was measured in short-term for both the control and combination groups with reference to the “New Standard for the Evaluation of the Efficacy of Tumor Immunotherapy” [10], and the total scores of the two observation points were compared at intervals of 4 weeks or more, according to the results of imaging examinations such as enhanced CT and MRI. The extent to which tumor burden increased or decreased from the baseline tumor burden and the effectiveness of therapy fall into one of four categories as follows: (1) complete remission (CR), in which all lesions have vanished; (2) partial remission (PR); (3) disease progression (PD): *a* > 20% increase in baseline tumor burden (total assessed tumor burden); and (4) Stable disease (SD): ineligible for CR and PR criteria without PD. The total effective rate of the two groups was calculated from the above four conditions (total effective rate = (CR + PR)/total number of cases × 100%), disease control rate (DCR) = (CR + PR + SD)/total number of cases × 100%.

#### 2.4.2. Scores of Changes in Quality of Life

The KPS score method was used for evaluation, and the changes in the KPS score of the two groups of patients were divided into 3 grades (1) improvement: KPS score after treatment increased by more than 10 points compared with before treatment; (2) stable: KPS score after treatment compared with before treatment, it increased or decreased by less than 10 points; (3) decreased: the KPS score after treatment decreased by more than 10 points compared with before treatment.

#### 2.4.3. Adverse Reactions

According to “CTCAE Version 5.0,” the adverse reactions of patients during treatment can be divided into grades 0-IV. In this study, the occurrence of adverse reactions such as proteinuria, fatigue, elevated alanine aminotransferase, hypertension, immune pneumonia, muscle and joint pain, and hypothyroidism was observed and recorded in the two groups.

### 2.5. Immune Function

Before and after treatment, flow cytometry was used to detect the changes in peripheral blood *T* cell subsets (CD3+, CD4+, CD8+, and CD4+/CD8+) and B lymphocyte (CD19+) levels in the two groups of patients.

### 2.6. Short-Term Survival Prognosis

After the treatment was completed, all patients were followed up by telephone. Follow-up was terminated when the patient's disease progressed or died or at the last follow-up, and the PFS of the two groups of patients was recorded.

### 2.7. Statistical Analysis

SPSS 19.0 was used to conduct the statistical analysis. Data from measurements are presented as means ± standard deviations (*X* ± SD), and differences between groups were determined using a two-sample*t*-test; data from counts are reported as percentages of a given population (n), and differences between the two groups were determined using a *χ*^2^ test. For this study, a probability of 0.05 or less indicated statistical significance.

## 3. Result

### 3.1. Clinical Data Comparison

Forty individuals, ages ranging from 45 to 86, made up the final control group. Patients' ages ranged from 44 to 87 in the combined group. Patients in both groups were comparable with regard to age, KPS score, gender, smoking history, and the occurrence of extrathoracic metastases (*P* > 0.05; Table 1).

### 3.2. The Short-Term Treatment Effect of the Two Groups of Patients

The overall efficiency of the control group was 27.50% and the combined group was 48.89%. It was determined that there was a statistically significant difference between the two groups (*χ*^2^ = 4.0790, *P* < 0.05). The disease control rate of the control group was 72.50%, which was statistically (*χ*^2^ = 6.6810, *P* < 0.05) lower than the treatment disease control rate (93.33%) of the combination group. The particular details are given in Table 2.

### 3.3. KPS Scores of the Two Groups of Patients

The increase of the KPS score in the combined group was 91.11%, much greater than that of the control group 42.50% (the difference was statistically significant; *χ*^2^ = 23.0900, *P* < 0.05). The details were shown in Table 3.

### 3.4. Before and after Immunotherapy in the Two Groups of Patients

Pretreatment CD3+, CD4+, CD19+, CD8+, and CD4+/CD8+ cell counts were similar between the two groups (*P* > 0.05). Post-treatment, the combination group had significantly higher CD3+, CD4+, and CD4+/CD8+ ratios than the control group (*P* < 0.05). CD8+ and CD19+ cell counts did not vary significantly between the combination and the control groups (*P* > 0.05, Table 4).

### 3.5. The Adverse Reactions of the Two Groups of Patients

Standard adverse events occurred with similar frequency in both groups (all *P* > 0.05). Proteinuria, weariness, increased alanine aminotransferase, hypertension, immunological pneumonia, musculoskeletal pain, and hypothyroidism were the most common adverse effects in both groups. The grade IV adverse reactions were very uncommon (Table 5).

### 3.6. PFS Analysis of the Two Groups of Patients

**Figure 1:**
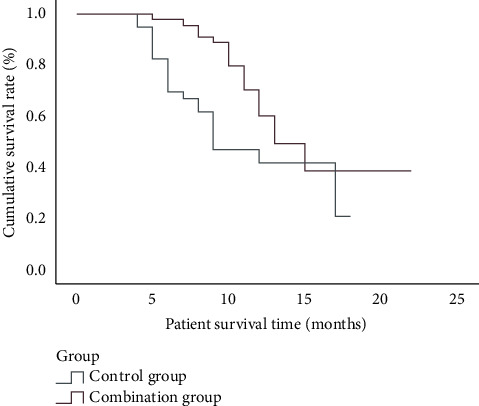
Survival analysis curve.

After follow-up until March 2022, the median PFS of the control group was (9.00 ± 1.65) months (95% CI: 5.76–12.24). The median PFS of the combined group was (13.00 ± 1.10) months (95% CI: 10.84–15.16), and the difference was statistically significant (*P* < 0.05, Table 6, Figure 1).

## 4. Discussion

The incidence of lung adenocarcinoma, a common pathological subtype of lung cancer, is rising in recent years. It is not uncommon for lung adenocarcinoma to be discovered at a late stage after the greatest window of opportunity for surgical intervention has passed, since its early signs are not visible. The primary goals of treatment are survival extension, symptom relief, and enhanced quality of life.

Immune checkpoint inhibitors have shown long-lasting responses in some patients with advanced lung adenocarcinoma, and this has led to a dramatic shift in the therapeutic landscape. Compared to placebo plus pemetrexed and platinum, first-line pembrolizumab with pemetrexed plus platinum substantially improved overall survival (OS) and PFS in patients with metastatic nonsquamous NSCLC in the clinical trial KEYNOTE-189. In patients with advanced NSCLC who had progressed after platinum-based chemotherapy, the combination of pembrolizumab and docetaxel was well tolerated and significantly improved ORR and PFS in a randomized phase 2 clinical trial named PROLUNG [11]. In non-squamous non-small-cell carcinoma, the combination of the immune checkpoint inhibitor PD-1 immune drug pembrolizumab and bevacizumab was recently approved, and it reduced the risk of progression by 38% compared with bevacizumab combined with chemotherapy. This finding opened the door to new combination therapy approaches [12]. The immune checkpoint inhibitor PD-1 is a human immunoglobulin G4 monoclonal antibody that can bind to its receptor, thereby blocking the inhibitory effect of the PD-1 signaling pathway on immune cells and enhancing its immunity to tumor cells, which in turn leads to tumor necrosis and infiltration of mononuclear cells into the tumor.

In 2020, the Chinese Society of Clinical Oncology (CSCO) guidelines on non-small-cell lung cancer proposed using first-line antiangiogenic therapy for stage IV nonsquamous NSCLC without a driver gene. Based on the BEYOND study conducted in the Chinese population, the median PFS of bevacizumab combined with platinum doublet group prolonged by 2.7 months compared with the simple chemotherapy group (9.2 months vs 6.5 months, HR = 0.40, *P* < 0.001). The median OS was significantly prolonged to 24.3 months (24.3 months vs. 17.7 months) and the ORR is also significantly improved (52% vs. 26%, *P* < 0.001) with good safety. The guideline regards bevacizumab combined with platinum-containing doublet chemotherapy + bevacizumab maintenance therapy as a class I recommendation (category 1A and 2A evidence). These findings reflect the status of bevacizumab in the treatment of advanced lung adenocarcinoma. The formation of new blood vessels plays a very important role in the occurrence, development, and metastasis of tumors. Bevacizumab can normalize abnormal new blood vessels by inhibiting angiogenesis, thereby achieving the purpose of inhibiting tumor growth and metastasis. Moreover, bevacizumab could enhance the therapeutic efficacy of immune checkpoint inhibitors, and play a synergistic effect in combination with immune checkpoint inhibitors. The mechanism of action of bevacizumab is that the immune checkpoint inhibitor PD-1 is a human immunoglobulin G4 monoclonal antibody, which blocks the programmed death protein 1 (PD-1) of T cells and the programmed death protein (PD) expressed by tumor cells. PD ligand (PD-L1) binding or cytotoxic T lymphocyte-associatedprotein-4 (CTLA-4) binding to CD80 (B7-1) activates tumor immune responses, but only about 20–30% of patients receive long-lasting effects with a single immunotherapy drug because cancer cells use a variety of ways to block the immune response and lead to immune escape. The reason is the hypoxic immunosuppressive microenvironment formed by abnormal tumor proliferation and angiogenesis. Angiogenetic factors promote tumor angiogenesis under hypoxia and down-regulate the immune function of the body. Antiangiogenic therapy can activate immunotherapy by inducing the normalization of tumor blood vessels, increasing immune cell infiltration in tumor tissue, and reducing the immunosuppressive state. However, Allen et al. [13] observed in tumor models that after antiangiogenetic treatment, the expression of PD-L1 in tumors increased, and the combination with PD-1 on the surface of T cells caused T cells to inactivate or die and inhibited tumor immunity. Therefore, the addition of anti-PD-L1 therapy can block the combination of the two, enhancing and prolonging the efficacy of antiangiogenic drugs on malignant tumors. The combination therapy of these two types of drugs can induce the normalization of tumor blood vessels, reverse the inhibitory immune microenvironment of tumors, and synergistically synergize, showing good efficacy and drug safety, and ultimately benefiting patients' survival. Regarding the clinical efficacy of antiangiogenesis combined with immune check inhibitors on patients, Wang et al. [14] have reported relevant research results. The survival of 67 patients with advanced NSCLC who had previously received anti-PD-1 drugs (pembrolizumab, nivolumab, carrelizumab) in combination with anlotinib was analyzed. The results showed that ORR and DCR were 28.4% and 86.6%, respectively, suggesting that anlotinib combined with anti-PD-1 drug therapy can benefit these patients. The results suggest that anlotinib combined with anti-PD-1 therapy has tolerable toxicity and favorable antitumor activity in previously treated patients with advanced NSCLC. This result added to the growing evidence supporting the use of immunotherapy. The benefits of combining antiangiogenic drugs.

In relapsed and newly diagnosed patients, inhibition of VEGF with bevacizumab in NSCLC significantly prolonged PFS, but not OS. VEGF inhibition can enhance immunotherapeutic benefits in a variety of cancers through multiple mechanisms and preclinical studies. This result has also been confirmed in this study. Pembrolizumab with bevacizumab improves overall treatment efficiency and disease control rates in patients with driver gene-negative stage IV lung adenocarcinoma compared to pembrolizumab monotherapy. The data show that the combination of bevacizumab and pembrolizumab may boost the short-term effectiveness of therapy for stage IV lung cancer. Patients with advanced lung cancer benefited from PD-1 coupled with bevacizumab, as shown by the research by Ferrara et al [15].

The immune functional status of patients with lung adenocarcinoma is critical throughout the occurrence and development of the disease and is closely related to the patient's treatment response and survival prognosis. In tumor patients, tumor cells interact with immune cells to jointly promote the occurrence and development of tumors. This tumor-specific immune response can be recorded in a timely manner by the body. It can accurately reflect the status of genes, which plays a significant guiding role in the systematic evaluation of the individual's immune status, tumor occurrence, development, prognosis, and guidance of treatment. The level of T cell subsets is an important indicator to reflect the body's cellular immune function, and immune function plays an important role in the disease progression of patients with malignant tumors. When patients suffer from tumors, cancer cells will weaken the body's immune function, and a series of toxic effects produced by chemotherapeutic drugs during the treatment period will also damage the body's immune system, resulting in reduced immunity of patients, which is not conducive to the recovery of the disease. CD3+ participates in T cell signal transduction, which reflects the overall cellular immune function status of the body. CD4+ recognizes antigen signal transduction, and has auxiliary and amplifying effects on the production of antibodies, the activation of macrophages, and cytotoxic T lymphocytes [16]. The decrease in the number of CD4+ molecules represents the decrease in immune function. CD8+ is a kind of suppressor T cell that functions as a killer cell during an immune response. The CD4+/CD8+ ratio may be used as an indicator of the health of cells in the immune system. B cell proliferation, differentiation, activation, and antibody production are all influenced by CD19+, a membrane antigen. Results demonstrated that there were no significant differences (*P* > 0.05) in the number of pretreatment CD3+, CD4+, CD19+, CD8+, or CD4+/CD8+ counts between the two groups. Following treatment, both CD8+ and CD19+ counts were considerably higher than those in the control group (*P* < 0.05), although there was no statistical difference between the two groups (*P* > 0.05). Under normal circumstances, the ratio of CD4+/CD8+ is in a relatively balanced state to maintain the normal immune response of the body. When the ratio decreases, it means that the cellular immune function of the body is reduced, the killing effect of tumor cells is weakened, and the patient's body is in an immunosuppressive state. In the study, the CD3+, CD4+, and CD4+/CD8+ index values were significantly increased after treatment compared with those before treatment. It shows that the treatment of patients with bevacizumab combined with pembrolizumab may activate most of the T lymphocytes and significantly improve the cellular immune function of the patient, independent of the immune regulation of B cells. Analysis of the reasons, the upregulation of angiogenic growth factor expression can reduce the function of T cells in the tumor microenvironment, thereby inhibiting the antitumor immune response, while bevacizumab treatment can promote the transport and infiltration of T cells, thereby enhancing the antitumor immunity in the tumor microenvironment. The combination of immune checkpoint inhibitor therapy can significantly improve the patient's immune function, and maximize the benefits of patient survival. Given that bevacizumab improves PFS in patients with advanced NSCLC adenocarcinoma, including bevacizumab in the research may increase pembrolizumab's exposure and hence, its potential therapeutic efficacy. There has been growing evidence from both domestic and international research in recent years demonstrating the importance of measuring the ratio of T lymphocyte subsets in determining lung cancer prognosis and gauging the success of treatment. Per Luo et al. [17], high levels of infiltration by + *T* cells, CD8+ T cells, Foxp3+ T cells, and their corresponding normal tissues have been demonstrated to correlate negatively with the prognosis of cancer patients. Hiroyasu [18] reported that recurrence-free survival was significantly lower in the high FOXP3+/CD4+ cell ratio group than in the low FOXP3+/CD4+ cell ratio group. FOXP3+/CD4+ cell ratio can be used to predict the prognosis of lung adenocarcinoma. Our results are similar to those of this researcher.

In addition, the rate of growth and stability of the KPS score was much greater and the progression-free survival time was significantly longer in the combination group compared to the control group. Proteinuria, tiredness, increased alanine aminotransferase, hypertension, immunological pneumonia, muscle pain, arthralgia, and hypothyroidism were the most common adverse effects in both groups, with grades I, II, and III being the most common and degree IV being uncommon. There was no statistically significant difference in the frequency of adverse responses between the groups, demonstrating that pembrolizumab and bevacizumab together are well tolerated and did not exacerbate adverse reactions in patients. But from the detailed data, it seems that the control group had a little greater rate of adverse responses than the combination group and that the combination group needed to be monitored for hypertension and proteinuria. Overall, the quality of life of patients was enhanced and their median overall survival time was extended when the immune checkpoint inhibitor pembrolizumab was combined with bevacizumab [1].

## 5. Conclusion

In conclusion, this study demonstrates that combining bevacizumab with pembrolizumab for the treatment of patients with stage IV lung adenocarcinoma considerably increases the short-term therapeutic effectiveness, enhances immune function, improves the quality of life, and extends PFS of patients.

## Figures and Tables

**Table 1:** The clinical information of the two patient groups.

Item	Control group (*X *± SD/*n*(%))	Combination group (*X *± SD/*n*(%))	*t*/*χ*^2^	*P*
Age (Years)	66.30 ± 9.42	65.69 ± 9.64	0.2914	0.7714

KPS Score (Points)	44.98 ± 9.47	45.38 ± 9.89	0.1889	0.8506

Gender				
Male	27 (67.50)	25 (55.56)	1.2720	0.2594
Female	13 (32.50)	20 (44.44)	-	-

Smoking history				
Yes	35 (87.50)	42 (93.33)	0.8452	0.3579
No	5 (12.50)	3 (6.67)	-	-

Extrathoracic metastases				
Head metastases	9 (22.50)	11 (24.44)	0.0445	0.8329
Bone metastases	2 (5.00)	4 (8.89)	0.4882	0.4847
Lever metastases	2 (5.00)	3 (6.67)	0.1063	0.7445
Other	1 (2.50)	0 (0.00)	1.1380	0.2860

PD-L1Expression				
< 1%	4 (10.00)	6 (13.33)	0.2267	0.6340
1–49%	29 (72.50)	32 (71.11)	0.0202	0.8871
50–100%	5 (12.50)	7 (15.56)	0.1631	0.6863
Not clear	2 (5.00)	0 (0.00)	2.3040	0.1290

**Table 2:** The curative effect of patients in the control group and combination group after treatment (*n*, (%)).

Group	Treatment efficacy				Total efficacy	DCR
	CR	PR	PD	SD		
Control group	3 (7.50)	8 (20.00)	11 (27.50)	18 (45.00)	11 (27.50)	29 (72.50)
Combination group	9 (20.00)	13 (28.89)	3 (6.67)	20 (44.44)	22 (48.89)	42 (93.33)
*χ * ^2^	2.7290	0.8995	6.6810	0.0026	4.0790	6.6810
*P*	0.0985	0.3429	0.0097	0.9590	0.0434	0.0097

**Table 3:** KPS scores of patients in the control group and combination group (*n*, (%)).

Group	KPS score			Increase stability
	Improve	Stabilized	Decrease	
Control group	11 (27.50)	6 (15.00)	23 (57.50)	17 (42.50)
Combination group	24 (53.33)	17 (37.78)	4 (8.89)	41 (91.11)
*χ * ^2^	5.8350	5.5670	23.0900	23.0900
*P*	0.0157	0.0183	< 0.0001	< 0.0001

**Table 4:** Immune cell status before and after treatment in the control group and combination group (*X* ± SD).

Group	Time	CD3^+^	CD4^+^	CD19^+^	CD8^+^	CD4^+^/CD8^+^
Control group	Before treatment	53.88 ± 6.78	30.30 ± 4.01	12.83 ± 2.63	28.60 ± 3.35	1.07 ± 0.15
	After treatment	50.53 ± 10.64	28.15 ± 3.91	12.70 ± 2.70	28.30 ± 2.94	1.01 ± 0.22

Combination group	Before treatment	54.04 ± 6.66	30.53 ± 4.78	12.38 ± 2.98	28.87 ± 3.21	1.08 ± 0.24
	After treatment	65.29 ± 9.04 ^*∗*^	37.04 ± 4.18 ^*∗*^	12.82 ± 3.10	28.89 ± 3.26	1.30 ± 0.22 ^*∗*^

*Note. * ^*∗*^*P* < 0.05, compared with the control group.

**Table 5:** The adverse effects of the therapy in the control group and the combination group (*n*, %).

Adverse effect	Control group			Combination group		
	I/II	III/IV	Total incidence	I/II	III/IV	Total incidence
Proteinuria	0 (0.00)	0 (0.00)	0 (0.00)	1 (2.22)	0 (0.00)	1 (2.22)
Fatigue	19 (47.50)	3 (7.50)	22 (55.00)	16 (35.56)	1 (2.22)	17 (37.78)
Elevated ALT	13 (32.50)	0 (0.00)	13 (32.50)	15 (33.33)	1 (2.22)	16 (35.56)
Hypertension	0 (0.00)	0 (0.00)	0 (0.00)	1 (2.22)	0 (0.00)	1 (2.22)
Immune pneumonia	3 (7.50)	0 (0.00)	3 (7.50)	4 (8.89)	0 (0.00)	4 (8.89)
Muscle and arthralgia	6 (15.00)	0 (0.00)	6 (15.00)	4 (8.89)	1 (2.22)	5 (11.11)
Hypothyroidism	3 (7.50)	0 (0.00)	3 (7.50)	3 (6.67)	0 (0.00)	3 (6.67)

*Note.* ALT is alanine aminotransferase.

**Table 6:** PFS analysis of the two groups of patients.

Group	Median PFS	95% CI	Average PFS	95% CI	*P value*
Control group	9.00 ± 1.65	5.76∼12.24	11.48 ± 0.91	9.69∼13.26	0.020
Combination group	13.00 ± 1.10	10.84∼15.16	15.52 ± 0.88	13.79∼17.25	

## Data Availability

All experimental data used to support the findings of this study are available from the corresponding author upon request.
